# Retraction: Synthesis of non-toxic, biocompatible, and colloidal stable silver nanoparticle using egg-white protein as capping and reducing agents for sustainable antibacterial application

**DOI:** 10.1039/d2ra90063g

**Published:** 2022-06-17

**Authors:** Kalaiyarasan Thiyagarajan

**Affiliations:** Defence Institute of High Altitude Research (DIHAR), Defence Research and Development Organization (DRDO), C/o 56 APO Leh-Ladakh-194101 India advances-rsc@rsc.org +91-172-2638900 +91-172-2642900

## Abstract

Retraction of ‘Synthesis of non-toxic, biocompatible, and colloidal stable silver nanoparticle using egg-white protein as capping and reducing agents for sustainable antibacterial application’ by Kalaiyarasan Thiyagarajan *et al.*, *RSC Adv.*, 2018, **8**, 23213–23229, https://doi.org/10.1039/C8RA03649G.

I, the undersigned author, hereby wholly retract this *RSC Advances* article due to the following instances of matched/similar images that have been identified that weaken this article, which occurred due to honest human errors.

Following the previous publication of a correction to replace Fig. 3D, instances of duplicating images have been identified that undermine this article.

In Fig. 5, the panels *E. coli* MTCC No 62 6 μg ml^−1^ and *E. coli* MTCC No 62 8 μg ml^−1^ are identical. Furthermore, they are identical to panels in another article published by the authors in *RSC Advances*,^1^ namely the panel for freshly prepared *S. enterica* MTCC-3219 2 μg ml^−1^ 10^−10^ and the panel for *S. typhirmurium* MTCC-3224 4 μg ml^−1^ 10^−5^ after one year of storage in Fig. 3 of ref. 1 and the panel 1 h treated with SBT@AgNPs in Fig. 7 of ref. 1.

Thiyagarajan Kalaiyarasan and Vijay K. Bharti responded to all enquiries and submitted data related to the above concern. However, to avoid any future ambiguity to the readers, the article is retracted.

Krishna Kumar and Vijay K. Bharti do not agree to the retraction. The other authors have been informed but have not responded to any correspondence regarding the retraction.

Signed: Kalaiyarasan Thiyagarajan

Date: 1/6/2022

Retraction endorsed by Laura Fisher, Executive Editor, *RSC Advances*

## Supplementary Material
